# Bioluminescence based biosensors for quantitative detection of enterococcal peptide–pheromone activity reveal inter-strain telesensing *in vivo* during polymicrobial systemic infection

**DOI:** 10.1038/srep08339

**Published:** 2015-02-09

**Authors:** Sabina Leanti La Rosa, Margrete Solheim, Dzung B. Diep, Ingolf F. Nes, Dag Anders Brede

**Affiliations:** 1 Department of Chemistry, Biotechnology and Food Science, Norwegian University of Life Sciences, Aas, Norway

## Abstract

*Enterococcus faecalis* is a significant threat in the nosocomial setting due to the emergence of isolates that are multi-antibiotic resistant, refractory to the available therapies and equipped with a variety of pathogenicity determinants. This bacterium uses quorum-sensing systems to regulate its physiological processes, including the expression of virulence traits, to adapt and proliferate within a host. Here, we describe the construction and application of two bioluminescence-based reporter systems for the direct detection of the quorum-sensing regulated expression of (i) the gelatinase biosynthesis-activating pheromone (GBAP) and (ii) the cytolysin small subunit (CylL_S_) in natural samples. The two *E. faecalis* reporters conditionally expressed bioluminescence in the presence of GBAP and CylL_S_ both in the supernatants of liquid cultures and in an agar-overlay assay in as little as three hours, with a high level of sensitivity. Biosensors employed to investigate the interaction between the *fsr* and *cyl* systems revealed that *fsr* impeded CylL_S_ activity by 75%. Furthermore, we identified a clinical *E. faecalis* isolate that acted as a biological cheater, producing cytolysin only upon sensing CylL_S_-producers in its environment. This isolate enhanced its virulence during polymicrobial systemic infection of *Galleria mellonella*.

Bacterial telesensing is guided by small extracellular molecules that are produced and secreted to actively query the environment^1^. Concentration or physiochemical changes in those ‘probes' are perceived as discharging environmental signals, allowing the bacteria to trigger an appropriate response in the host. Responses, such as enhancing their fitness for the colonisation of an environmental niche via subversion of the host's immune responses, releasing virulence factors as a defensive mechanism against competitive microorganisms or eukaryotic cells, differentiating into certain morphological forms in hostile environments and regulating genetic exchange, are coordinated through the telesensing systems^1^,^2^. The best characterised form of telesensing is quorum sensing (QS), in which bacteria sense and adapt to environmental conditions by coordinating and adjusting gene expression according to the local population density^3^.

*Enterococcus faecalis* is a gram-positive bacterium that commonly dwells in the gastrointestinal tract of healthy humans, animals and insects^4^,^5^. Although some strains have been safely used for decades as probiotics^6^, *E. faecalis* has rapidly emerged as a causative agent of hospital-acquired infections worldwide^7^. The spread of this opportunistic pathogen has been facilitated by its ability to tolerate and adapt to many types of environmental stresses and acquire high-level resistance to commonly used antibiotics^8^,^9^,^10^,^11^. The pathogenicity of *E. faecalis* has been linked to its production of factors involved in cell and tissue damage, adherence to cells and extracellular surface proteins, and evasion of the host immune system^12^. Three prominent peptide pheromone systems associated with highly virulent strains of *E. faecalis* are involved in environmentally regulated telesensing systems, including a conjugative system mediated by pheromone-responsive plasmids, the Fsr regulatory system, and cytolysin signalling^13^,^14^,^15^.

Cytolysin is a two-peptide lantibiotic haemolytic toxin of *E. faecalis* that requires the expression of two divergently organised multicistronic operons localised either on pheromone-responsive plasmids or on a pathogenicity island^13^,^16^. Two promoters, P_Lys_ for the structural genes and P_Reg_ for two regulators, control the expression of the *cyl* locus. Mature cytolysin consists of two peptides—CylL_S_ and CylL_L_—and acts as a cytolytic toxin through forming a complex in eukaryotic and prokaryotic cellular membranes that leads to membrane rupture. Once synthesised, the two precursors are post-translationally modified by the product of the gene *cylM* and are secreted into the extracellular environment by the CylB transporter. Outside the cell, they eventually undergo a proteolytic activation through the action of CylA. In the absence of target cells, the larger subunit, CylL_L_, forms a stable inactive complex with the small subunit CylL_S_ and inhibits its cytolytic activity. However, in the presence of a target, CylL_L_ binds with a higher affinity to the cellular lipid membrane than to CylL_S_. As a consequence, the locally accumulating free mature CylL_S_ will reach a certain threshold concentration that leads to the de-repressed binding of the regulatory proteins CylR1 and CylR2 on P_Lys_ and the autoinduction of the cytolysin operon. An additional gene, *cylI*, provides self-protection from the bactericidal effect of the toxin. Through this regulatory system, *E. faecalis* can therefore finely tune the expression of *cyl* genes according to the presence of target cells.

The Fsr system is a major virulence regulator in *E. faecalis* and comprises four genes that are responsive to the extracellular accumulation of the gelatinase biosynthesis-activating pheromone (GBAP)^15^,^17^. The P*_fsrB_* promoter initiates the transcription of an operon comprising three genes: *fsrB*, *fsrD* and *fsrC*. The *fsrD* gene encodes the precursor of GBAP, which is processed by the product of *fsrB* following its extracellular release. The local accumulation of the GBAP peptide is sensed by the histidine kinase FsrC, which is on the surface of *E. faecalis*, which then activates the response regulator FsrA by phosphorylation. FsrA acts as a transcription factor that up regulates expression through the P*_fsrB_* promoter as well as through a promoter that controls the coordinate expression of two virulence factors, the gelatinase GelE and the serine protease SprE.

In a recent study, our group constructed and assessed the use of two bioluminescence-based systems for the *in vivo* non-invasive monitoring of *E. faecalis* cytolysin- and gelatinase-promoter activity in the murine intestine and during the systemic infection of *Galleria mellonella* larvae and mice^18^. By determining the bioluminescence emission at different time points during the progression of an *E. faecalis* infection, we showed that both the gelatinase and cytolysin promoters were subjected to temporal regulation and that the expression of these traits was controlled in response to sensing diverse environmental conditions.

In this study, we describe the use of two bioluminescence-based reporter systems as biosensors for the direct detection and quantification of GBAP and CylL_S_ in biological samples. The two biosensors are based on the P*_gelE_* and *cyl*R2R1P*_cyl_* promoters that drive the GBAP- or CylL_S_-induced expression of the *luxABCDE* operon specifically in the presence of true pheromone producers. Our results showed that the biosensors are suitable for the rapid, sensitive and real-time detection of positive isolates directly in natural samples and demonstrated for the first time that enterococcal telesensing both *in vitro* and *in vivo* during polymicrobial infection in *G. mellonella* larvae is possible.

## Results and Discussion

### Development of *E. faecalis* biosensors for detection of CylL_S_ and GBAP pheromones

In a previous study, we utilised *E. faecalis* variants that expressed the *luxABCDE* cassette under the control of the cytolysin or gelatinase promoter to monitor the expression of the two factors during *in vitro* growth and during the infection of the mouse model. The results showed that the *fsr* and *cyl* QS- reporter systems were both modulated by environmental cues^18^.

In the current study, we constructed and employed *lux*-based biosensors for the detection and quantification of the cytolysin subunit CylL_S_ and the gelatinase biosynthesis-activating pheromone (GBAP) of *E. faecalis*^19^,^20^.

The cytolysin biosensor was constructed by introducing the CylL_S_-responsive regulatory genes and the *cyl* promoter onto a *lux*-containing vector. The resulting plasmid, pSL101*cylR2R1*P*_cyl_*, was introduced into *E. faecalis* JH2-2^18^, a plasmid-free derivative of *E. faecalis* JH2, which lacks the *cyl* operon and is therefore unable to produce or sense the toxin^21^. For simplicity, in the following text, we will refer to the cytolysin biosensor as JH2-2 CBS.

**Figure 1:**
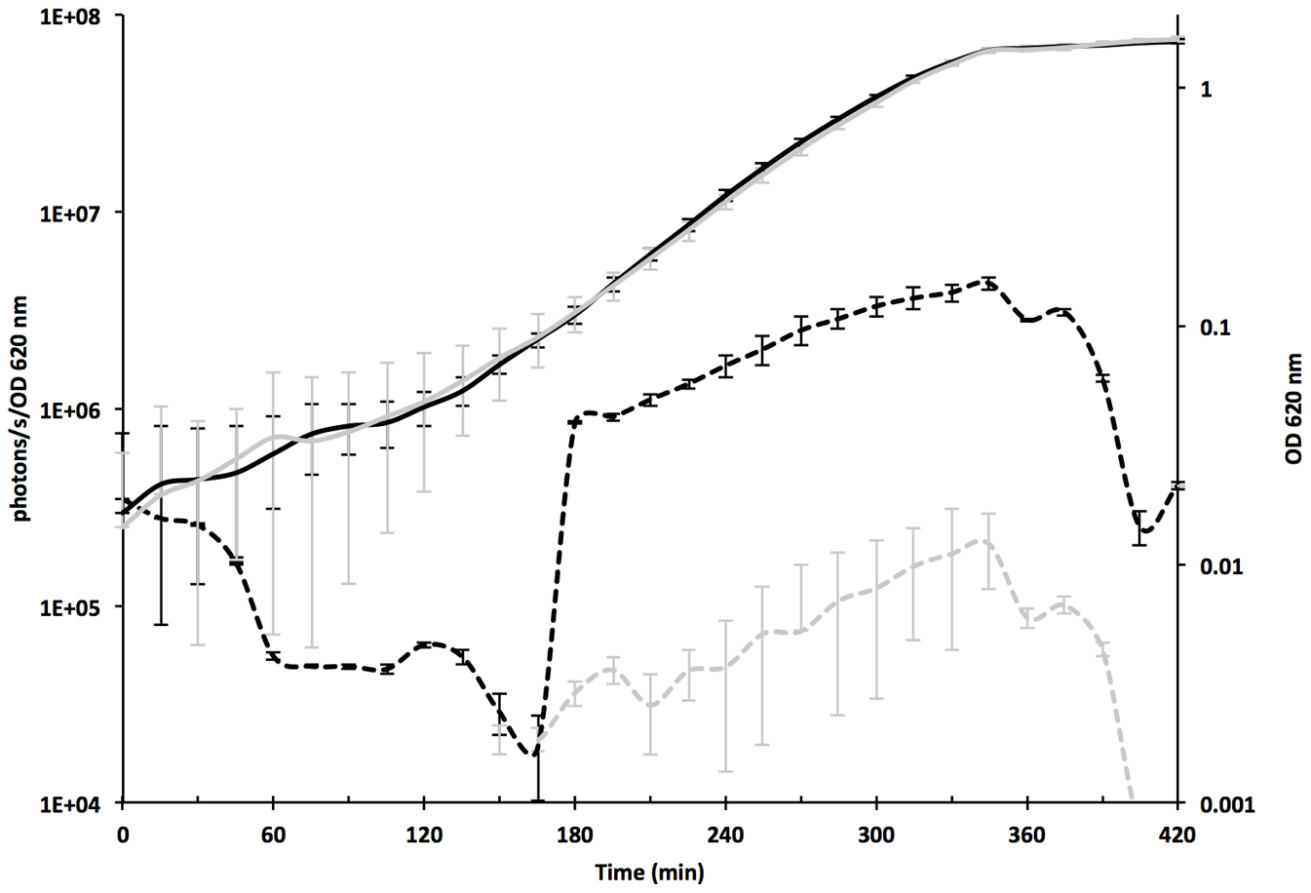
Performance of the P*_gelE_* and P*_fsrB_* promoters during the growth of *E. faecalis* SL11 (MMH594::pREG696*lux*P*_gelE_*, in black) and SL13 (MMH594::pREG696*lux*P*_fsrB_*, in grey) in GM17 medium. The dotted lines indicate the promoter activity expressed as bioluminescence (photons/s) divided by the optical density (OD) at 620 nm, and growth is indicated by continuous lines and was measured as OD_620_. The values shown represent the averages of the results of three biological replicates ± standard deviation.

To develop a functional GBAP biosensor system, we investigated the expression profile of both *fsrB* and *gelE* promoter-driven *lux* expression in *E. faecalis* MMH594. The growth of SL11 and SL13, which are, respectively P*_fsrB_* and P*_gelE_* reporter strains, at 37°C in GM17 medium was compared by monitoring the optical density at 620 nm. No significant difference in the growth rate or the final cell density was observed. This result indicated that the presence of the biosensor system did not hamper normal cell growth (Figure 1). Both promoters led to the emission of bioluminescence throughout growth, with similar expression patterns; in both strains, the signal was low during the early exponential phase and increased from the beginning of the mid-logarithmic phase, before waning during the stationary phase. Nevertheless, under the condition of equal amounts of pheromone, P_f*srB*_ showed a lower level of specific activity, with only a 10-fold increase, whereas P*_gelE_*-driven *luxABCDE* expression increased 230-fold during growth. These results are consistent with the outcomes of previous studies showing that the phosphorylated response regulator FsrA had a lower binding affinity for the *fsrB* promoter than for the *gelE* promoter^15^,^22^. Due to the higher level of expression driven by the P*_gelE_* promoter under the conditions tested, pREG696 *lux*P*_gelE_* was therefore selected for introduction into *E. faecalis* V583fsrB* for use as the GBAP reporter (aka V583fsrB* GBS).

### Biosensor proof of concept: Screening CylL_S_ and GBAP production by genome-sequenced *E. faecalis* isolates

**Figure 2:**
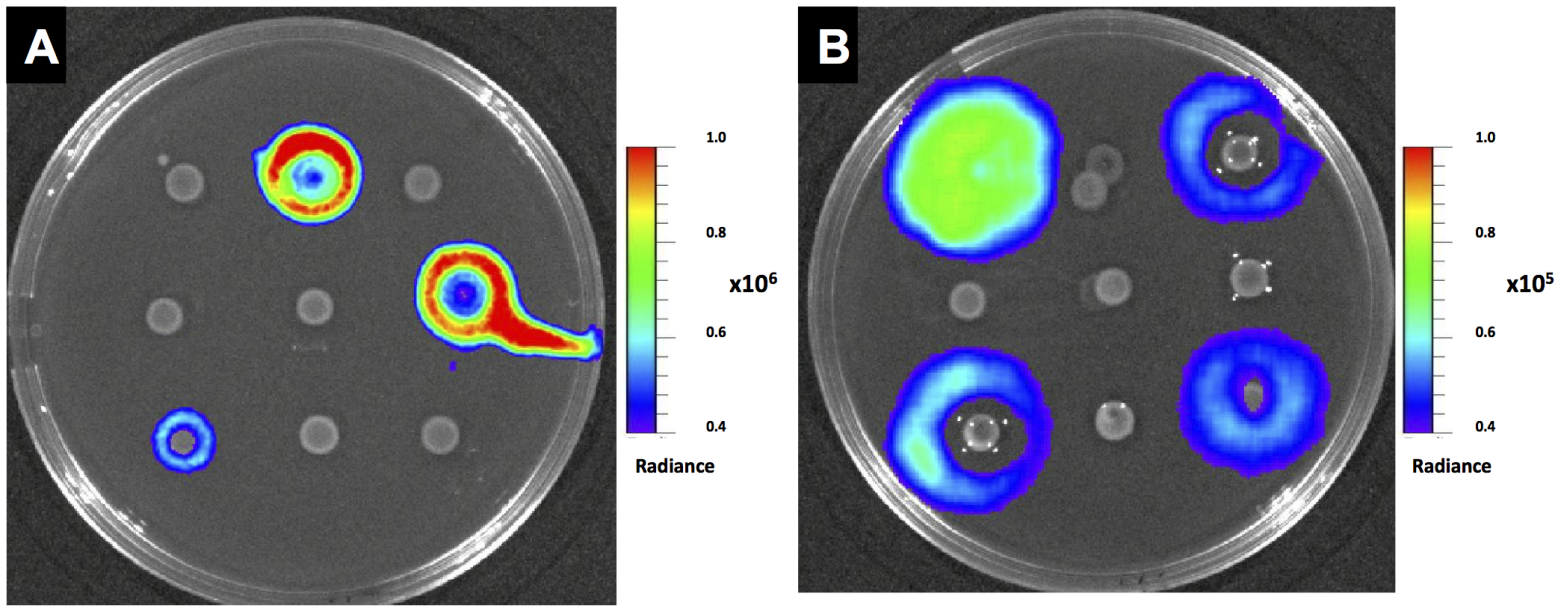
Bioluminescence-based detection of CylL_S_ (A) and GBAP (B) producers. From the right to the left, uppermost row: V583, DS5, and E1Sol; central row: V583fsrB*, CH188, and ×98; bottom row: MHH594, T2, and V583Δ*gelE*. The *E. faecalis* isolates were cultured on GM17 agar plates, and the biosensor was overlaid after an overnight incubation. Plates were kept at 37°C for 3 hours and imaged with a Xenogen IVIS Lumina II Imaging System (Calipers Corp., CA). A 10-fold higher light emission was induced by CylL_S_ than by GBAP.

To test the ability of the JH2-2 CBS and V583fsrB* GBS to sense the presence of CylL_S_ and GBAP producers in the environment, we employed nine genome-sequenced *E. faecalis* strains of clinical and commensal origin and developed a screening method that utilised GM17-agar plates. The strains had been previously tested for their cytolytic phenotype and GBAP-production ability (S. Leanti La Rosa, L. G. Snipen, B. E. Murray, R. Willems, M. S. Gilmore, D. B. Diep, I. F. Nes, and D. A. Brede, submitted). The panel included the cytolysin-positive strains DS5 and ×98, the GBAP-positive isolates E1Sol, V583 and V583Δ*gelE*, the GBAP- and cytolysin-producer MMH594, the GBAP- and cytolysin-negative strains T2 and CH188, and V583fsrB*, which harbours a mutation in the *fsrB* gene and is thus unable to synthesise the GBAP pheromone. Cells of the above-mentioned strains were cultured on two GM17-agar plates that were individually overlaid with the biosensors. Induction of visible light emission by the CylL_S-_ and GBAP-producers but not by the non-producers occurred by 3 hours following the application of the appropriate biosensor (Figure 2). These bioluminescently tagged *E. faecalis* strains may therefore offer a simple, cost-effective and rapid method for determining the presence of cytolysin or GBAP producers in food, water, faecal and clinical samples. Furthermore, the systems were highly specific in sensing CylL_S_ and GBAP and therefore would effectively prevent the false positive assumption of virulence traits based on only the detection of genes that might not necessarily lead to the corresponding phenotype.

**Figure 3:**
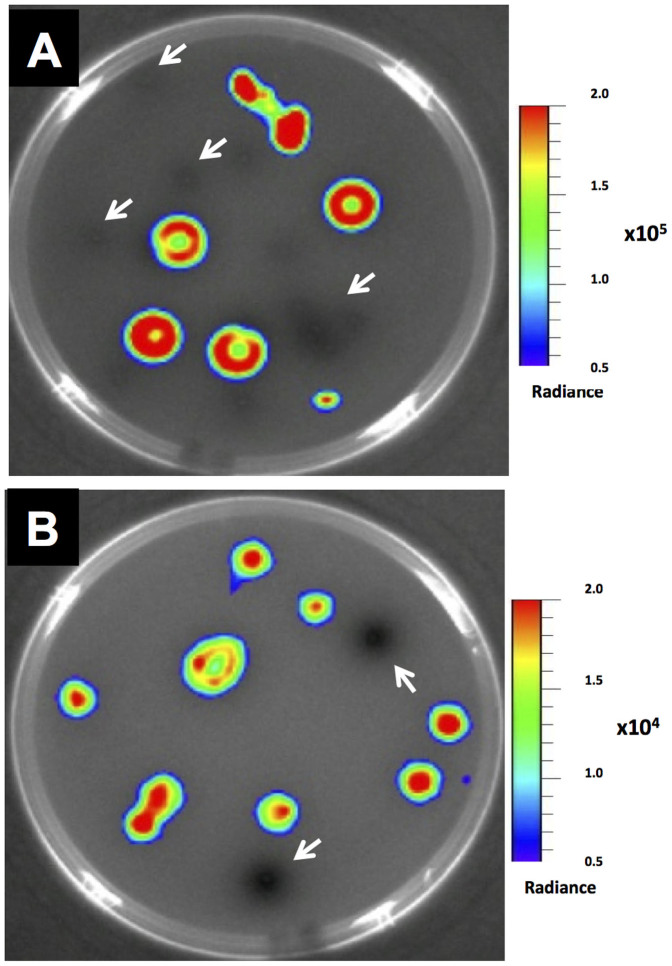
Bioluminescence imaging of CylL_S_ (A) and GBAP (B) producers on BEA. The white arrows indicate cells that were unable to produce CylL_S_ (A) or GBAP (B). After overnight incubation at 37°C, plates seeded with a mixed population of CylL_s_ and GBAP producers were overlaid with the JH2-2 CBS (A) or V583fsrB* GBS (B), following additional incubation for 3 hours. Imaging was performed with a Xenogen IVIS Lumina II Imaging System (Calipers Corp., CA).

To assess the feasibility of the biosensor systems to directly detect cytolysin- and GBAP-positive strains in natural samples growing on enterococci-selective media, mixed cultures of clinical *E. faecalis* isolates were plated on bile esculin agar (BEA) and overlaid with JH2-2 CBS or V583fsrB* GBS biosensors. In both cases, the biosensor overlay allowed the identification of bioluminescence-inducing colonies in as little as three hours, confirming that the screening method was effective in detecting a specific producer within a sample and is suitable for real-time monitoring of pathogens without the need for pure cultures (Figure 3).

### The JH2-2 CBS and V583fsrB* GBS detected high levels of CylLs- and GBAP-pheromone activities in cell-free culture supernatants

**Figure 4:**
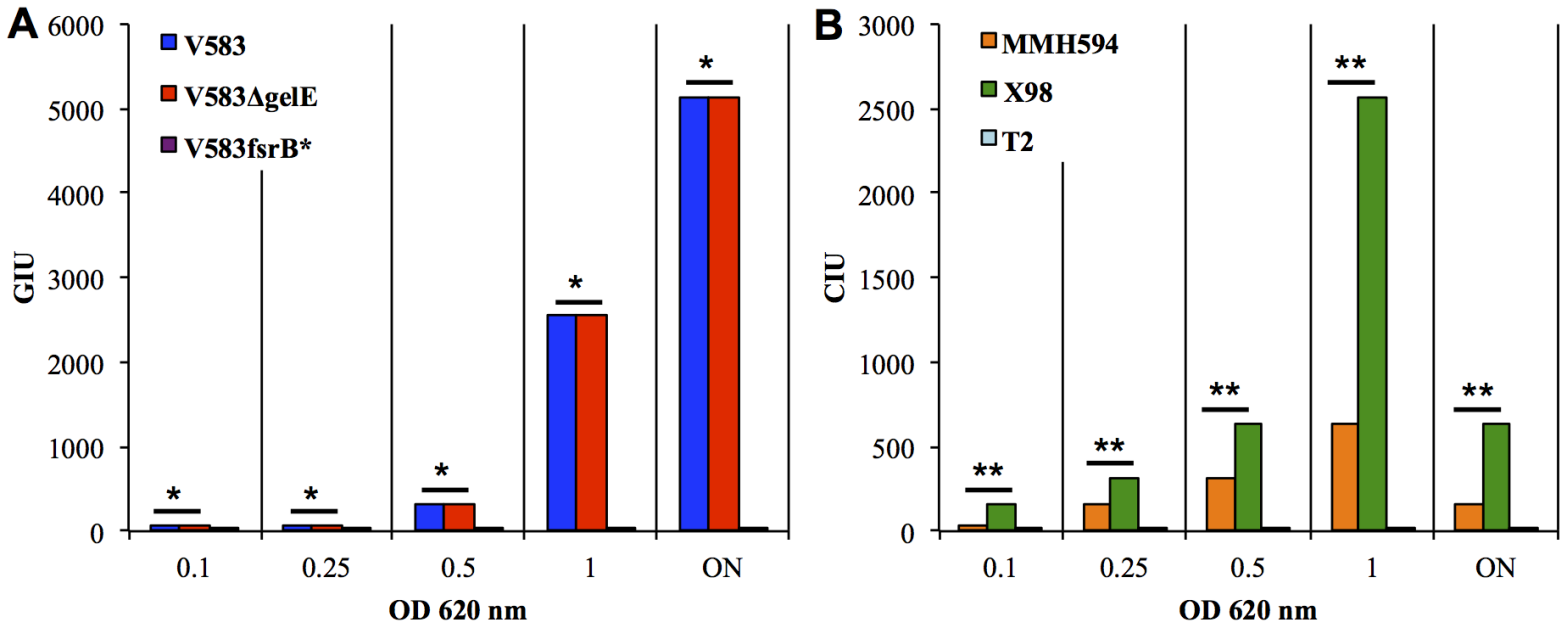
(A). Determination of GBAP-Inducing Units in an overnight (ON) culture and during the growth (at increasing OD_620_ units) of a culture of *E. faecalis* V583 wt (blue) or V583Δ*gelE* (red) V583fsrB* (violet) did not give rise to a detectable signal. (B). Determination of CylL_S_-Inducing Units during the growth of a culture and in an overnight (ON) culture of *E. faecalis* ×98 (green) and MMH594 (orange). The addition of the JH2-2 CBS to the T2 (light blue) supernatant did not give rise to a detectable signal at any dilution. GIU, GBAP inducing units; CIU, CylL_S_ Inducing Units. * p-value > 0.05. ** p-value < 0.05.

To corroborate the applicability of the CylL_S_ and GBAP biosensors, we employed them for the direct detection and quantification of the two pheromones in liquid substrates. Whereas GBAP has been successfully isolated from the supernatants of *E. faecalis* cultures^17^, previous studies reported that cytolysin was clearly produced by *E. faecalis* growing on blood agar or when erythrocytes were added to broth cultures but a low level of activity was observed in liquid cultures^23^,^24^. It was therefore of interest to determine whether the CylL_S_ and GBAP biosensors were able to specifically sense the local accumulation of mature pheromones in culture supernatants. To investigate GBAP detection, serial dilutions of supernatants from overnight and exponential-phase cultures of *E. faecalis* V583, V583Δ*gelE* and V583fsrB* were mixed with an equal amount of a 50-fold dilution of the GBAP biosensor in its exponential-phase. Bioluminescence was detected in the supernatants of the *E. faecalis* V583 and V583Δ*gelE* cultures but not in those of the V583fsrB* cultures, confirming the specificity of the construct, which reacted only to the gelatinase biosynthesis-inducing pheromone (Figure 4A). A highly significant correlation was found between the level of light emission in photons/s over a three-fold order of serial dilution of the samples of the supernatants of exponential- (R^2^ > 0.90) and stationary-phase cultures (R^2^ > 0.96), showing that this biosensor is an accurate and sensitive mean for quantifying the concentration of the GBAP pheromone. The measured GBAP activity was 320 GIU in exponential phase supernatants and 5120 GIU in overnight culture supernatant (data not shown). GBAP was detected at similar levels in the supernatants of both V583 and V583Δ*gel*E stationary- and exponential-growth phase cultures (P > 0.05, according to the Mann-Whitney test). This finding suggested that the gelatinase of these isolates did not inactivate or affect the biological activity of GBAP. The high levels of GBAP activity observed in the overnight cultures strongly suggested that the down-regulation of the *fsr* circuit during the stationary phase was not due to the inactivation of GBAP activity.

Similarly, the JH2-2 CBS facilitated the specific detection of active CylL_S_ in cell-free supernatants. JH2-2 CBS sensed up to 640 CIU in the supernatants of cells in the exponential phase of growth and 5120 CIU in overnight culture supernatant (data not shown). The exhibited levels of bioluminescence showed a linear dose-response dependency with the CylL_S_ concentration (R^2^ > 0.97). This trend allowed the quantitative measurement of CylL_S_ activity during bacterial growth. CylL_S_ was found to accumulate in large amounts during the exponential growth phase of the CylL_S_-producers ×98 and MMH594 but was found to be totally absent in cultures of the non-producer T2 (Figure 4B). To the best of our knowledge, the presence of a high level of biologically active CylL_S_ pheromone in the absence of target cells has not been reported hitherto. Surprisingly, the content of CylL_S_ increased during the growth of *in vitro* monocultures, despite the fact that CylL_S_ and CylL_L_ are known to form non-haemolytic oligomers^25^. This result implied that *cyl* signalling was highly responsive even when the level of toxin activity was low.

### Impairment of cytolysin production is mediated by the metalloprotease GelE

**Figure 5:**
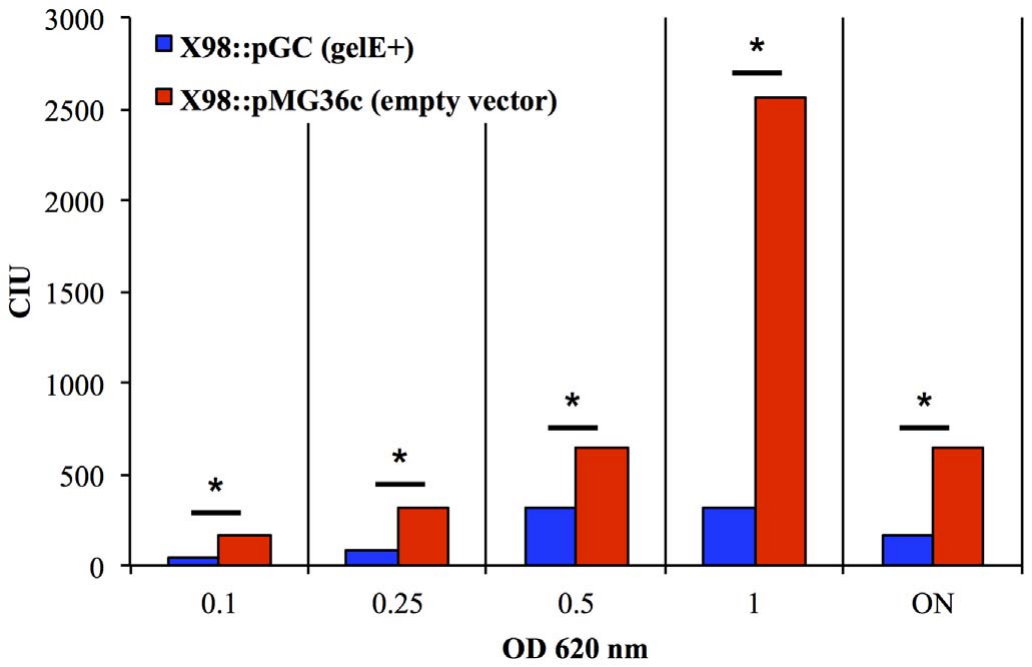
Determination of CylL_S_-Inducing Units (CIU) in overnight (ON) cultures and during the growth (at increasing OD_620_ units) of cultures of *E. faecalis* ×98 pMG36c (red; control) and ×98 pCG (blue; GelE-overexpressing strain). The plot displays the averages of the results of triplicate independent experiments. * p-value < 0.05.

Interestingly, we observed a 2-fold difference in the levels of CylL_S_ production in ×98 and MMH594 exponential-phase cultures, and a 4-fold higher level of CylL_S_ in the supernatants of late exponential- and stationary-phase cultures of ×98 compared with those of MMH594 (P < 0.001, according to the results of the Mann-Whitney test) (Figure 4). In a recent study in which we investigated the pathogenicity of *E. faecalis* in the nematode *Caenorhabditis elegans*, statistical analyses showed that the concomitant expression of both gelatinase and cytolysin through the *E. faecalis* genome significantly increased its virulence (S. Leanti La Rosa, L. G. Snipen, B. E. Murray, R. Willems, M. S. Gilmore, D. B. Diep, I. F. Nes, and D. A. Brede, submitted). Interestingly, the effect of the co-presence of these two virulence traits was much less than the sum of their main individual effects, described as saturation or antagonistic effects. It was previously reported that GelE contribute to the virulence of *E. faecalis* by triggering the proteolytic degradation of a broad range of host substrates^26^,^27^,^28^, and *gelE* expression was found to have a profound effect on the secretome of *E. faecalis*^29^,^30^. GelE activity was found to be required for a variety of processes, such as the regulation of the display of the surface adhesin Ace^31^ and the activation of the primary autolysin AtlA and its contribution to biofilm formation^32^. Additionally, the production of gelatinase was reported to impair other cellular activities, such as conjugation due to the degradation of sex pheromone-related peptides^33^. Taken together with the results of utilising the CylL_S_-biosensor detection system, we considered the possibility that gelatinase might also have an impact on cytolysin. To test this hypothesis, we employed the JH2-2 CBS biosensor to detect CylL_S_ accumulation in the supernatants of exponential- and stationary-phase cultures of the gelatinase-overproducing *E. faecalis* strain ×98::pCG (Figure 5). Under both of the tested conditions, our data showed that introducing gelatinase into the Cyl producer ×98 leads to a 4- to 8-fold reduction of CylL_S_ activity (p < 0.05, according to the results of Mann-Whitney test).

We previously observed that cytolysin is expressed mostly during the late exponential phase and that its expression gradually subsides during the stationary phase^18^. This trend is consistent with a scenario in which GelE is able to degrade part of the CylL_S_ that was produced during the active growth phase and that GelE continued to exert its proteolytic activity long after the production of CylL_S_ subsided. The fact that Cyl production was reduced by 75% was consistent with our previous observations regarding the pathogenicity of *E. faecalis* in *C. elegans* (S. Leanti La Rosa, L. G. Snipen, B. E. Murray, R. Willems, M. S. Gilmore, D. B. Diep, I. F. Nes, D. A. Brede, submitted). It thus appears probable that this phenomenon was highly effective *in vivo*.

### Interstrain CylL_S_-mediated communication demonstrated the occurrence of an *in vivo* cheating behaviour

Quorum sensing is a specific type of telesensing that allows cell-cell communication via small diffusible molecules that are released to explore the surrounding environment^34^. In its simplest form, quorum sensing enables the bacteria to control the production of molecules that are released into the extracellular environment and become available not only for sibling producer cells but also for any other cells present. However, it has been reported that individuals that do not respond to quorum-sensing signals act as cheaters, not incurring the metabolic cost of producing those released molecules while benefiting from those secreted by cooperators^35^. The cheater gains a fitness advantage over the quorum-sensing positive strains. The production of extracellular metabolites, such as virulence factors, may lead to the recognition of the bacteria and ultimately destruction by the host immune system^36^. Therefore, quorum sensing can be used to trigger concerted gene activation in the microbial community to effectively respond to the prevailing conditions, which could be activating processes for evading a host or escaping the host's defences.

**Figure 6:**
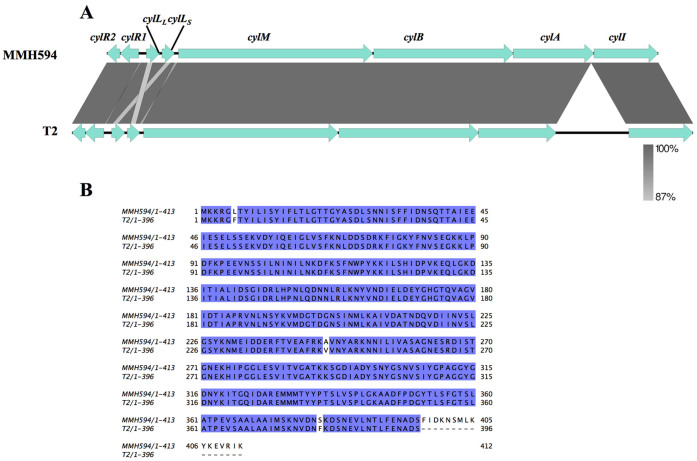
(A) Alignment of the *cyl* operons of *E. faecalis* MMH594 and T2. A BlastN comparison was performed using EasyFig version 2.1 software. The levels of similarity ranged from 100 to 87%, as shown in the grey gradient scale. The light blue arrows indicate the genes. (B) Comparison of CylA sequences of MMH594 and T2. The conserved residues are highlighted in blue. The alignment was conducted using the MAFFT alignment program.

Studies have shown that the *cyl* locus is subject to genetic instability both *in vitro* and *in vivo*^37^,^38^. A number of investigators have reported phenotypic instabilities with no obvious explanations at the genetic level, indicating that silent or non-functional *cyl* genes occur in the genomes of enterococcal isolates of different origins^39^,^40^,^41^. In a previous report, we identified an apparent incongruence between the *cyl* genotype and phenotype in the clinical isolate T2^42^ (S. Leanti La Rosa, L. G. Snipen, B. E. Murray, R. Willems, M. S. Gilmore, D. B. Diep, I. F. Nes and D. A. Brede, submitted). Here, we performed a detailed comparative sequence analysis of the *cyl* locus of a subset of genome-sequenced *E. faecalis* strains, including the strain MMH594, and we detected the presence of an IS6770 element that was integrated into the 3′-end of the *cylA* gene in T2 (Fig. 6A). The insertion causes a premature stop and the production of a truncated, and presumably non-functional, CylA (Fig. 6B).

**Figure 7:**
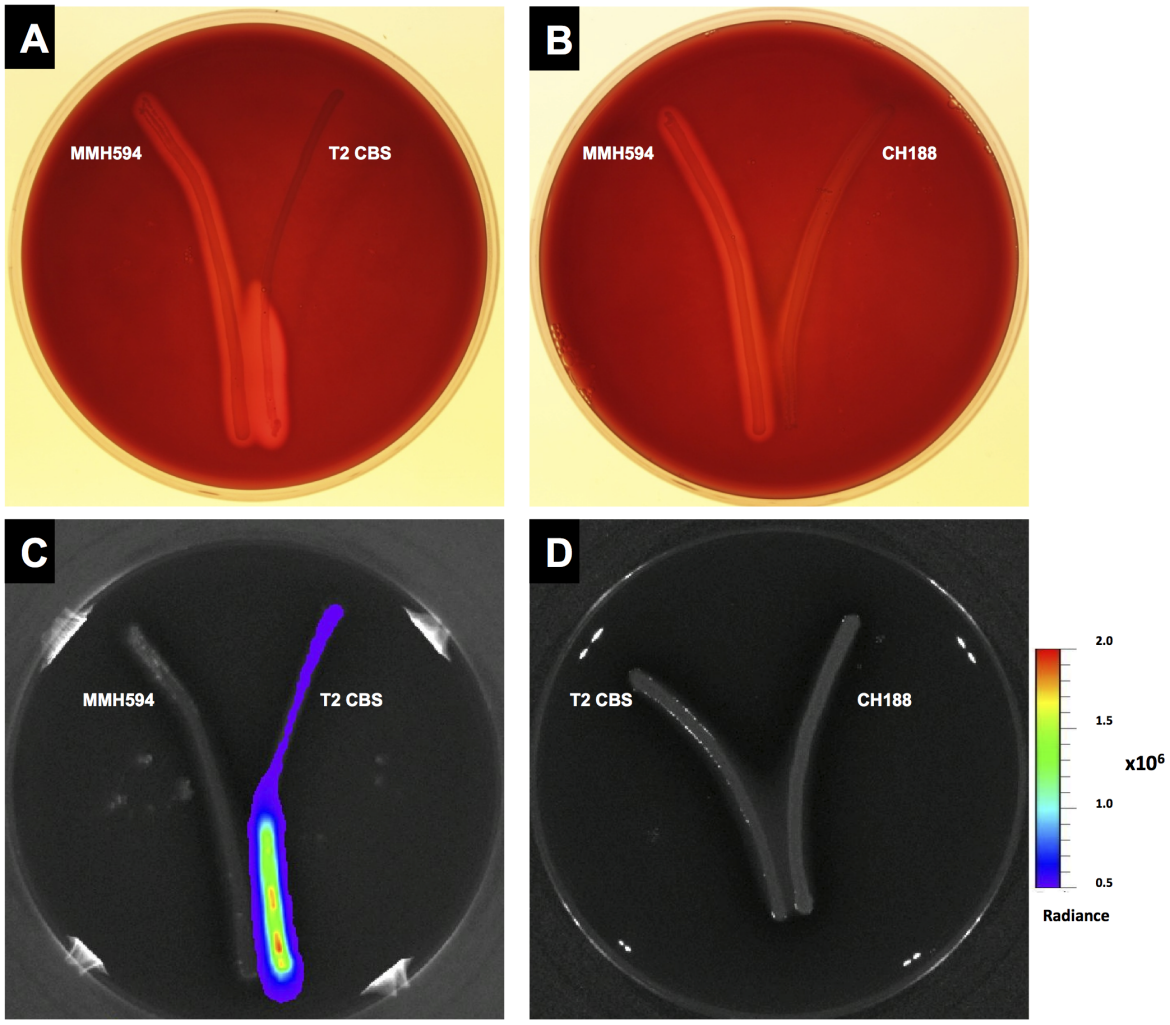
*E. faecalis* interstrain communication. Cultivating *E. faecalis* T2 CBS in proximity to MMH594 induced cytolysin production by T2, visible as a clear zone, (A) and bioluminescence emissions that propagated to the top of the streak (C), whereas no induction was detected when T2 CBS was cultivated near CH188 (D). The control isolate CH188 maintained its cytolysin-negative phenotype when cultivated in proximity to MMH594 (B). Experiments were performed on BHI plates supplemented with 1% (v/v) defibrinated horse blood, 1% (w/v) glucose and 0.03% (w/v) L-arginine.

We then tested the hypothesis that *E. faecalis* strains exhibiting a cytolysin-negative phenotype but harbouring the elements of the *cyl* locus could act as social cheaters that cease production of quorum-controlled cytolysin and benefit from or take advantage of the release of CylL_S_ by cooperators. A variant of *E. faecalis* T2 tagged with pSL101*cylR2R1*P*_cyl_* (named T2 CBS) was employed; the assessment of cytolysin production mediated by sensing the local accumulation of mature CylL_S_ subunits in the environment was performed by growing *E. faecalis* T2 CBS in proximity to the cytolysin-positive strain MMH594 on blood-agar plates (Figure 7). A diffuse and bright haemolytic zone was observed in the area in which the two strains were in proximity to each other (Fig. 7A) indicating that *E. faecalis* T2 CBS was able to detect and use the CylL_S_ released by the MMH594 cells to trigger its own cytolysin production. Imaging analysis showed the ‘flare'-like induction of bioluminescence throughout the streak of T2 CBS (Figure 7C). Haemolysis or the ‘flare' effect was not detected when JH2-2, a strain lacking all of the genes necessary for cytolysin production, was streaked near T2 CBS cells (data not shown). In this case, bioluminescence arose only from the JH2-2 CBS cells in close proximity to MMH594 cells. No increase in the level of haemolysis or light emissions was observed when MMH594 or T2 CBS cells were cultivated in proximity to *E. faecalis* CH188, which lacks a complete *cyl* operon^43^ (Figures 7B and 7D). In addition, no haemolysis was observed when T2 CBS was cultivated alone or in proximity to its parental strain on blood-agar plates (data not shown). To test whether the cytolysin-positive phenotype was stably imposed on the T2 cells, cells that had been induced once were re-streaked on blood agar. However, these cells consistently reverted to a cytolysin-negative phenotype (data not shown). Based on these results, we hypothesised that *E. faecalis* T2 acted as a social cheater, adopting the strategy of reducing its metabolic burden by avoiding the production and release of the toxin and benefiting from a growth advantage over cytolysin-producing cells. However, this strain retained its immunity to the bactericidal effect and its ability to exploit the release of CylL_S_ into the environment by a primary producer to efficiently spread during a polymicrobial infection.

### *In vivo* cytolysin telesensing during infections of *G. mellonella* larvae exacerbated the virulence

**Figure 8:**
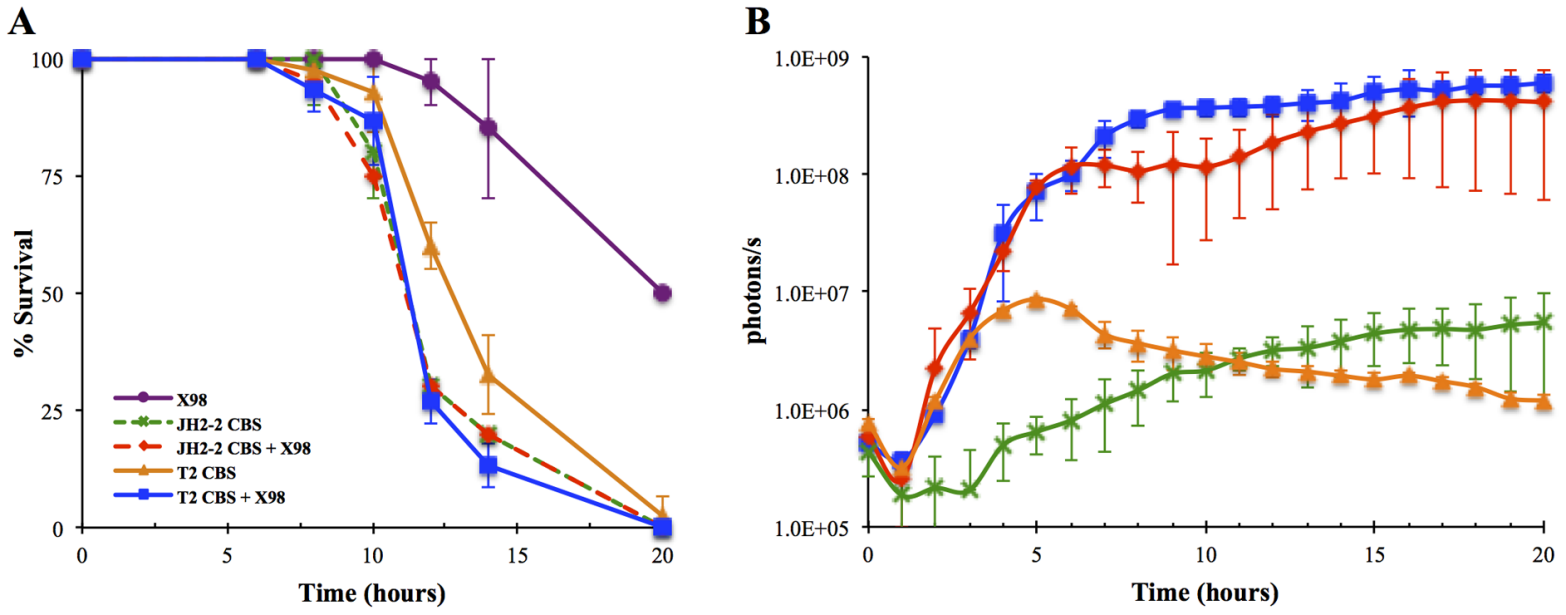
*G. mellonella* larvae were injected by *E. faecalis* ×98 (purple), T2 CBS (orange), JH2-2 CBS (green), and a mixed suspension of either ×98 + T2 CBS (blue) or ×98 + JH2-2 CBS (red). (A). Survival curves of *G. mellonella* larvae following infection with *E. faecalis*. Co-infection by T2 CBS and ×98 caused increased killing to that resulting by T2 CBS mono-infection, (P = 0.015, chi-square test with 1 degree of freedom, T2 CBS + ×98 *versus* T2 CBS). The plots display the averages of results of triplicate independent experiments. For each assay, 10 insects were used. (B). Real-time monitoring of CylL_S_ expression during the progression of an *E. faecalis* infection in intact insects. For tracking bacterial infection in *G. mellonella*, five individual insects were injected and placed in duplicate in a 4.0 cm Petri dish. Plates were kept at 37°C in the chamber of the Xenogen IVIS Lumina II imaging system (Caliper Life Sciences, CA). Bioluminescence was recorded at 30 minutes intervals for 20 hours.

A previous study indicated that appropriate regulation of both the gelatinase and the cytolysin promoter occurred during the mono-infection of *G. mellonella* larvae with *lux*-tagged *E. faecalis* MMH594 variants and that these traits were conditionally induced in response to the insect haemocoel environment^18^. To investigate whether the interstrain communication observed in T2 CBS (as described above) could affect pathogenicity *in vivo*, we performed a polymicrobial infection of *G. mellonella* larvae with *E. faecalis* T2 CBS in combination with the cytolysin-producer ×98. Injecting 2 × 10^6^ T2 CBS cells resulted in an LT_50_ of approximately 13 hours (Figure 8A) and low levels of bioluminescence (Figure 8B). Injecting an equal amount of inoculum of T2 CBS in combination with ×98 at a dose of approximately 2 × 10^5^ cells into the haemocoel of the insects resulted in an increased level of virulence, with an LT_50_ of 11 hours (P = 0.015, chi-square test with 1 degree of freedom, T2 CBS + ×98 *versus* T2 CBS). Moreover, the 100-fold increase in bioluminescence observed at 6 hours post infection was indicative of interstrain CylL_S_ communication. To explore the possibility that the increased pathogenicity could be due to the presence of ×98 or was a result of induced cytolysin production by T2, a similar experiment was performed using the JH2-2 CBS instead of the T2 CBS, because the JH2-2 strains lacks the *cyl* operon. In this experiment, the JH2-2 CBS demonstrated CylL_S_ interstrain communication, with a level of bioluminescence induction similar to that observed when T2 was used as the biosensor (Figure 8B), but there was no increase in the level of virulence (Figure 8A). Collectively, these results suggested that *in vivo* telesensing was a credible scenario during polymicrobial enterococcal infections that potentially could contribute to increasing the severity of such infections. These findings might have broad implications because a number of apparently *cyl*-negative phenotypic isolates contain intact *cyl* loci or a deletion in the *cylA* proteinase gene^40^,^41^. It is conceivable that such strains would both be immune to the cytolysin toxin and demonstrate cytolytic activity when sharing a habitat with a CylA-expressing strain. Alternatively, clinical isolates such as T2, which bears defective *cyl* loci, might have evolved during infection via a mechanism similar to that by which V583 descended from V586^44^.

Using bioluminescence imaging, we were able to follow the real-time dynamics of the CylL*_S_* levels in intact insects during a mortality assay (Figure 8B). No light was detectable at any time point in the larvae infected with ×98, which confirmed the absence of any background bioluminescence. A basal level of light emission was observed in the insects that had been injected with T2 CBS; however, the level of bioluminescence reached a peak at 5 h post infection and diminished progressively. Despite this basal signal, a 716-fold increase in the level of bioluminescence was observed in the larvae that had been co-infected with both isolates. The signal caused by infection with the mixed culture of T2 CBS and ×98 was 60-fold higher than the basal signal; the signal progressively increased and peaked at 8 h post-infection, concomitant with when the insects began to die, and persisted at this level throughout the death of the wax moths. Similarly, co-infection with JH2-2 CBS and ×98 gave rise to a 69–fold higher bioluminescence emission than the basal signal from larvae injected with JH2-2 CBS (Figure 8B).

Taken together, the evident change in the levels of virulence and P*_cyl_*-driven *lux*ABCDE expression that was observed during *G. mellonella* co-infection supported the hypothesis that a small population of CylL_S_ producers can induce cytolysin synthesis by responsive cheaters during a polymicrobial infection.

## Conclusions

In this report, we describe the construction of two biosensors for the simple and rapid detection of CylL_S_ and the gelatinase biosynthesis-activating pheromone in *E. faecalis*. We showed that these reporters can be used for the real-time identification of pheromone producers both on agar plates and from culture supernatants and illustrated the applicability of the constructs to natural samples, alleviating the need for pure cultures. The biosensors were used to monitor the production of the pheromones GBAP and CylL_S_ during growth and they detected quantitative between-strain differences in CylL_S_ activity.

Pursuing this observation, we showed that gelatinase had an antagonistic activity toward cytolysin production, probably through its proteolytic degradation of the cytolysin-toxin subunits. To the best of our knowledge, this is the first example of an antagonistic interaction between two virulence traits that has been demonstrated in *E. faecalis*.

We also used the biosensors to elucidate a novel type of social cheating mechanism, which enabled conditional toxin production in a certain strain of *E. faecalis*. The production of cytolysin by the cheater strain was dependent upon its recognition of the accumulation of CylL_S_ produced by another *cyl*-expressing strain in its surrounding environment. The relevance of this mechanism was corroborated during the polymicrobial infection of the *G. mellonella* model system. Therefore, these reporter systems represent a powerful tool for studying *E. faecalis* pathogenicity, which will allow the population dynamics of cytolysin and GBAP producers to be monitored and will expand the current knowledge of the expression and functional activity of *E. faecalis* genes in microbe-host interactions.

## Methods

### Bacterial strains, plasmids and growth conditions

The bacterial strains used in this study are listed in Table 1. Unless otherwise indicated, the *E. faecalis* strains were routinely cultivated at 37°C, without agitation, in M17 broth (Oxoid LTD, UK) supplemented with 0.4% w/v glucose (GM17). The *E. coli* strains were grown at 37°C, with shaking, in Luria-Bertani (LB, Oxoid LTD, UK) broth. The following antibiotic concentrations were used for the enterococci: spectinomycin, 500 μg/mL and chloramphenicol, 20 μg/mL. The following antibiotic concentrations were used for the *E. coli*: spectinomycin, 200 μg/mL and chloramphenicol, 10 μg/mL.

The *E. faecalis* CylL_S_-biosensor strains (CBS) were constructed by introducing the pSL101*cylR2R1*P*_cyl_* vector into *E. faecalis* strains JH2-2 and T2^18^. To develop a GBAP biosensor (GBS), the following two vectors were employed: pREG696 *lux*P*_fsrB_*^45^, which contained a 480-bp segment of the upstream region and the ATG codon of *fsrB* fused to the *luxABCDE* cassette of pPL2 *lux* and the *axe*-*txe* stability module, and pREG696 *lux*P*_gelE_*^18^.

The vectors were propagated in *E. coli* GeneHogs, and the plasmid DNA was isolated using the E.Z.N.A. Plasmid Mini Kit I (Omega Bio-tek, USA) prior to transferring it into the *E. faecalis* strains via electro-transformation^46^. The transformants were selected using GM17 plates containing spectinomycin.

### Assessment of P*_fsrB_* and P*_gelE_* promoter activities during *in vitro* growth

The growth and the luminescence expression driven by the P*_fsrB_* and P*_gelE_* promoter of the SL11 and SL13 *E. faecalis* strains, respectively, were evaluated as described previously^47^. Briefly, *E. faecalis* overnight (ON) cultures were diluted 100-fold using GM17 medium, grown at 37°C until they reached an optical density at 620 nm of 0.2 and then diluted 100-fold again using fresh GM17 broth. A 300-μl aliquot of the culture was added to the wells of a 96-well plate (Nunc, Thermo Fisher Scientific, Denmark) and the plate was incubated at 37°C under static conditions in a Spectrostar Nano microplate reader (BMG Labtech). The absorbance at 620 nm was measured at 15-min intervals for 7 h. For the bioluminescence measurements, 300 μl of the same culture was added to the wells of a black 96-well plate (Nunc, Thermo Fisher Scientific, Denmark), which was incubated at 37°C under static conditions in the chamber of a Xenogen IVIS Lumina II Imaging System (Calipers Corp., CA). Luminescence was measured every 15 min for 7 h, with a binning factor of 16, F-stop of 1 and an exposure time of 1 minute.

To determine the specific promoter activities, the photons/second emissions of the different *lux*-tagged *E. faecalis* cultures were normalised according to their growth, as expressed as the optical density (OD) at 620 nm.

### Validation of the specificity of the CylL_S_- and GBAP-producing colony-screening assays

Single colonies of genome-sequenced *E. faecalis* isolates that are known to produce CylL_S_ and/or GBAP were cultured overnight in GM17 broth at 37°C. A 2.5-μl aliquot of each culture was spotted onto two GM17 agar plates, which were incubated for 16 h at 37°C. The plates were subsequently overlaid with 10 mL of GM17 soft agar (0.8% w/v agar) that had been tempered to 50°C and were seeded with 200 μl of an overnight biosensor culture. After incubation at 37°C for 3 h, the plates were visualised using a Xenogen IVIS Lumina II Imaging System (Calipers Corp., CA), using a binning factor of 16, F-stop of 1 and an exposure time of 1 minute.

### Identification of CylL_S_ and GBAP producers on enterococci-selective plates

A mixed culture containing two known CylL_S_ and GBAP producers was plated on two bile-aesculin agar (BEA, Oxoid LTD., UK) plates, which were incubated at 37°C overnight. The dilution factor of the culture was adjusted so that the plating resulted in the production of 20–30 colonies per plate. After performing the biosensor overlay as described above, the plates were maintained at 37°C for 3 hours and were visualised using a Xenogen IVIS Lumina II Imaging System (Calipers Corp., CA), using a binning factor of 16, F-stop of 1 and an exposure time of 3 minutes.

### Detection of CylL_S_ and GBAP in *E. faecalis* supernatants

CylL_S_ and GBAP detection in the supernatants of cultures of genome-sequenced *E. faecalis* producers was performed using the biosensors JH2-2 CBS and V583fsrB* GBS, respectively. Single colonies of *E. faecalis* ×98, MMH594, T2, V583, V583Δ*gelE* and V583fsrB* were inoculated into 5 mL of GM17 broth, and the cultures were incubated overnight at 37°C. The cultures were then diluted 1:100 using fresh GM17 broth and grown at 37°C; aliquots of the cultures were taken when the OD_620_ values were 0.1, 0.25, 0.5 and 1.0. After centrifugation at 10,000 × *g* for 10 minutes to remove the cells, the culture supernatants were collected and stored at 4°C until use. The supernatants were subjected to serial 2-fold dilutions in a volume of 100 μl/well in a 96-well plate (Nunc, Thermo Fisher Scientific, Denmark), and 100 μl of a 50× diluted exponential phase culture or an ON culture of the appropriate biosensor was added to each well. After incubation at 37°C for 3 h, the levels of bioluminescence emission were measured using a Xenogen IVIS Lumina II Imaging System, using a binning factor of 16, F-stop of 1 and an exposure time of 1 minute.

One CylL_S_ inducing unit (CIU) was defined as the reciprocal of the highest level of dilution of a sample that provided a 2-fold increase in the level of bioluminescence of a 0.2-mL aliquot of a JH2-2 CBS culture compared with the level of basal expression. Similarly, 1 GBAP-inducing unit (GIU) was defined as the reciprocal of the highest level of dilution of a sample that provided a 2-fold increase in the level of bioluminescence of a 0.2-mL aliquot of a 0.2 mL culture of V583*fsr*B* GBS compared with the level of basal expression.

### *In vitro* detection of cytolysin-mediated interstrain communication

The cytolysin-positive *E. faecalis* strain MMH594 was inoculated onto a blood-agar plate (Brain-heart infusion agar supplemented with 5% (v/v) defibrinated horse blood, 1% (w/v) glucose and 0.03% (w/v) L-arginine (Sigma-Aldrich)) by creating a curved streak that reached the centre of the plate. Samples of *E. faecalis* CH188 or T2 CBS, which exhibited a negative haemolytic phenotype, were similarly streaked on the right side of the plate, 2-mm from and not touching the MMH594 cells. As a control, a plate was similarly seeded with CH188 and T2 CBS cells. The plates were incubated at 37°C for 16 h under anaerobic conditions. Imaging of the plates was performed as described above.

### *In vivo* CylL_S_ telesensing during polymicrobial systemic infection of *G. mellonella using E. faecalis*

*G. mellonella* larvae were infected with *E. faecalis* as previously described, with some minor modifications^47^. Briefly, exponential-phase cultures of *E. faecalis* ×98 and T2 CBS grown in GM17 broth were washed three times using a sterile 0.9% saline solution and were brought to concentrations of 2 × 10^7^ ± 0.8 × 10^7^ CFU/mL and 2 × 10^8^ ± 1.8 × 10^8^ CFU/mL, respectively. For the purpose of co-infection, ×98 and T2 CBS cells were mixed in a ratio of 1:10 v/v. Larvae (weighing approximately 3 mg and approximately 3 cm in length) were injected through the left hindmost proleg with 10 μl of *E. faecalis* solution using a Hamilton 710SNR 100-μL syringe (Hamilton Company) fitted with a 30 G needle (BD Microlance 3). For each assay, 10 insects were used in triplicate and the experiment was independently repeated at least two times. The larval survival rate was determined at 20 hours after infection. For real-time visualisation of *E. faecalis* infection in *G. mellonella*, five individual insects were injected as described above and were placed in duplicate in a 4.0-cm Petri dish. The plates were incubated at 37°C in the chamber of a Xenogen IVIS Lumina II imaging system (Caliper Life Sciences, CA) and the bioluminescence emissions were recorded at 30-minute intervals for 20 hours. The LT_50_ values (time at which the lethality of 50% of the insects was reached during a 20-hour period) were employed to compare the level of infectivity of the inoculum.

## Author Contributions

D.A.B., S.L.L.R. and M.S. designed and performed the experiments and analysed the data; D.A.B., D.B.D. and I.F.N. contributed to reagents/materials/analytical tools. S.L.L.R. and D.A.B. wrote the paper. All of the authors reviewed the final manuscript.

## Figures and Tables

**Table 1:** Bacterial strains and plasmids used in this study

Name	Description^a^	Reference
***E. faecalis***		
DS5	Cytolysin-positive clinical isolate	48
E1Sol	Gelatinase-positive commensal strain	49
CH188	Gelatinase- and cytolysin-negative clinical isolate	43
X98	Cytolysin-positive isolate	50
JH2-2	Gelatinase- and cytolysin-negative laboratory strain	21
T2	Gelatinase- and cytolysin-negative clinical isolate	42
MMH594	Gelatinase- and cytolysin-positive clinical isolate	51
V583	Gelatinase-positive clinical isolate	44
V583fsrB*	*E. faecalis* V583 with an amber point mutation in the *fsrB* codon that causes the loss of GBAP production; gelatinase-negative phenotype	Leanti La Rosa et al, submitted
V583Δ*gelE*	*E. faecalis* V583 *gelE* mutant; produces GBAP but has a gelatinase-negative phenotype	45
SL11	*E. faecalis* MMH594::pREG696 *lux*P*_gelE_*	This study
SL13	*E. faecalis* MMH594::pREG696 *lux*P*_fsrB_*	This study
JH2-2 CBS	*E. faecalis* JH2-2::pSL101*cylR2R1*P*_cyl_*	This study
T2 CBS	*E. faecalis* T2::pSL101*cylR2R1*P*_cyl_*	This study
V583fsrB* GBS	*E. faecalis* V583 fsrB*::pREG696 *lux*P*_gelE_*	This study
X98 pMG36c	*E. faecalis* ×98:: pMG36c	This study
X98 pCG	*E. faecalis* ×98:: pCG	This study
***Plasmids***		
pSL101*cyl*R2R1P*_cyl_*	Spc^r^, contains the *axe-txe* cassette and the *luxABCDE* operon under the control of the P*_cyl_* promoter and the regulatory genes *cylR2* and *cylR1*	18
pREG696 *lux*P*_gelE_*	Spc^r^, contains the *gelE* promoter fused to the *luxABCDE* operon	18
pREG696 *lux*P*_fsrB_*	Spc^r^, contains the *fsrB* promoter fused to the luxABCDE operon	45
pMG36c	Cam^r^,	52
pCG	Cam^r^	53

^a^Spc^r^, spectinomycin resistance; Cam^r^, chloramphenicol resistance.
